# Supplementary material for: A Critical View of the Application of the APEX Software (Aqueous Photochemistry of Environmentally-Occurring Xenobiotics) to Predict Photoreaction Kinetics in Surface Freshwaters
Source: Molecules. 2019 Dec 18;25(1):9. doi: 10.3390/molecules25010009 (PMC7017383; doi:10.3390/molecules25010009)
Supplement: Supplementary file 1 [file molecules-25-00009-s001.zip › 3.2.4_gcc-4.4.0.zip!/3.2.4_gcc-4.4.0/3.2.4_gcc-4.4.0/doc/octave/html/interpreter/Calling-a-Function-by-its-Name.html]

Calling a Function by its Name - Untitled


Next: Evaluation in a Different Context,
Up: Evaluation


---

### 9.1 Calling a Function by its Name

The `feval` function allows you to call a function from a string
containing its name. This is useful when writing a function that needs to
call user-supplied functions. The `feval` function takes the name
of the function to call as its first argument, and the remaining
arguments are given to the function.

The following example is a simple-minded function using `feval`
that finds the root of a user-supplied function of one variable using
Newton's method.

```
     function result = newtroot (fname, x)
     
     # usage: newtroot (fname, x)
     #
     #   fname : a string naming a function f(x).
     #   x     : initial guess
     
       delta = tol = sqrt (eps);
       maxit = 200;
       fx = feval (fname, x);
       for i = 1:maxit
         if (abs (fx) < tol)
           result = x;
           return;
         else
           fx_new = feval (fname, x + delta);
           deriv = (fx_new - fx) / delta;
           x = x - fx / deriv;
           fx = fx_new;
         endif
       endfor
     
       result = x;
     
     endfunction
```

Note that this is only meant to be an example of calling user-supplied
functions and should not be taken too seriously. In addition to using a
more robust algorithm, any serious code would check the number and type
of all the arguments, ensure that the supplied function really was a
function, etc. See Predicates for Numeric Objects, for example,
for a list of predicates for numeric objects, and see Status of Variables, for a description of the `exist` function.

— Built-in Function: **feval** (name, ...)  
> Evaluate the function named name. Any arguments after the first
> are passed on to the named function. For example,
>
> ```
>           feval ("acos", -1)
>                => 3.1416
> ```
>
> calls the function `acos` with the argument -1.
>
> The function `feval` is necessary in order to be able to write
> functions that call user-supplied functions, because Octave does not
> have a way to declare a pointer to a function (like C) or to declare a
> special kind of variable that can be used to hold the name of a function
> (like `EXTERNAL` in Fortran). Instead, you must refer to functions
> by name, and use `feval` to call them.

A similar function `run` exists for calling user script files, that
are not necessarily on the user path

— Function File: **run** (f)  
— Command: **run** f  
> Run scripts in the current workspace that are not necessarily on the
> path. If f is the script to run, including its path, then `run`
> change the directory to the directory where f is found. `run`
> then executes the script, and returns to the original directory.
>
>
>
> **See also:** system.
